# Differences in the Renal Accumulation of Radiogallium-Labeled (Glu)_14_ Peptides Containing Different Optical Isomers of Glutamic Acid

**DOI:** 10.3390/molecules29173993

**Published:** 2024-08-23

**Authors:** Kazuma Ogawa, Kota Nishizawa, Kenji Mishiro, Masayuki Munekane, Takeshi Fuchigami, Hiroaki Echigo, Hiroshi Wakabayashi, Seigo Kinuya

**Affiliations:** 1 Institute for Frontier Science Initiative, Kanazawa University, Kakuma-machi, Kanazawa 920-1192, Ishikawa, Japan; mishiro@p.kanazawa-u.ac.jp; 2 Graduate School of Medical Sciences, Kanazawa University, Kakuma-machi, Kanazawa 920-1192, Ishikawa, Japanmunekane@p.kanazawa-u.ac.jp (M.M.); t-fuchi@p.kanazawa-u.ac.jp (T.F.); h-echigo1010@stu.kanazawa-u.ac.jp (H.E.); 3 Department of Nuclear Medicine, Institute of Medical, Pharmaceutical and Health Sciences, Kanazawa University, Takara-machi 13-1, Kanazawa 920-8641, Ishikawa, Japan

**Keywords:** kidney accumulation, bone imaging, glutamic acid, bone metastases, gallium

## Abstract

Acidic amino acid peptides have a high affinity for bone. Previously, we demonstrated that radiogallium complex-conjugated oligo-acidic amino acids possess promising properties as bone-seeking radiopharmaceuticals. Here, to elucidate the effect of stereoisomers of Glu in Glu-containing peptides [(Glu)_14_] on their accumulation in the kidney, the biodistributions of [^67^Ga]Ga-*N*,*N*′-bis-[2-hydroxy-5-(carboxyethyl)benzyl]ethylenediamine-*N*,*N*′-diacetic acid-conjugated (l-Glu)_14_ ([^67^Ga]Ga-HBED-CC-(l-Glu)_14_), [^67^Ga]Ga-HBED-CC-(d-Glu)_14_, [^67^Ga]Ga-HBED-CC-(dl-Glu)_14_, and [^67^Ga]Ga-HBED-CC-(d-Glu-l-Glu)_7_ were compared. Although the accumulation of these compounds in the bone was comparable, their kidney accumulation and retention were strikingly different, with [^67^Ga]Ga-HBED-CC-(d-Glu-l-Glu)_7_ exhibiting the lowest level of kidney accumulation among these compounds. Repeated d- and l-peptides may be a useful method for reducing renal accumulation in some cases.

## 1. Introduction

Bone scintigraphy using bone-seeking radiopharmaceuticals, such as [^99m^Tc]Tc-MDP, has been a longstanding and effective method for detecting bone metastases because of its high sensitivity [1,2,3]. [^99m^Tc]Tc-MDP is a multinuclear complex that consists of a bisphosphonate compound with a remarkable affinity for bone, which is coupled with ^99m^Tc, a gamma ray-emitting radionuclide used for imaging. In the case of [^99m^Tc]Tc-MDP, ^99m^Tc seamlessly coordinates with the bisphosphonate, serving as a carrier that specifically targets bone metastases [4].

In pursuit of advancing bone-seeking radiopharmaceuticals via innovative drug design, our and other research groups and have successfully synthesized and assessed stable radiometal complex-conjugated bisphosphonate compounds [5,6,7,8,9,10,11,12,13,14,15,16,17]. They exhibited superior pharmacokinetics as bone-seeking radiopharmaceuticals. Concurrently, the potential of acidic amino acid peptides, specifically oligo-aspartic acids and oligo-glutamic acid molecules, which exhibit a notable affinity for bone, was explored [18,19]. Importantly, we demonstrated that radiogallium complex-conjugated oligo-aspartic acids and radiogallium complex-conjugated oligo-γ-carboxyglutamic acid molecules containing 1,4,7,10-tetraazacyclododecane-1,4,7,10-tetraacetic acid (DOTA) or *N*,*N*′-bis-[2-hydroxy-5-(carboxyethyl)benzyl]ethylenediamine-*N*,*N*′-diacetic acid (HBED-CC) as a chelator, to obtain a stable gallium complex, possess promising properties as bone-seeking radiopharmaceuticals [20,21,22]. Moreover, radiogallium-labeled hybrid types of peptides (between tumor-targeting and bone-seeking oligo-aspartic acids molecules) were also developed [23,24,25].

In our investigations, the bone accumulation patterns were comparable among [^67^Ga]Ga-DOTA-(l-Asp)_14_, [^67^Ga]Ga-DOTA-(d-Asp)_14_, [^67^Ga]Ga-DOTA-(l-Glu)_14_, and [^67^Ga]Ga-DOTA-(d-Glu)_14_, as they exhibited a similar high affinity for hydroxyapatite. Conversely, a distinct order emerged regarding their kidney accumulation: [^67^Ga]Ga-DOTA-(l-Asp)_14_ = [^67^Ga]Ga-DOTA-(d-Asp)_14_ < [^67^Ga]Ga-DOTA-(d-Glu)_14_ < [^67^Ga]Ga-DOTA-(l-Glu)_14_ [21]. In addition, a recent study reported by our group revealed that [^67^Ga]Ga-HBED-CC-(l-Glu)_14_ exhibited a higher kidney accumulation than [^67^Ga]Ga-HBED-CC-(dl-Gla)_14_ [22]. That is to say, Glu peptides are more highly accumulated in the kidney than other acidic acid peptides. There could be differences in renal accumulation depending on the optical activity of Glu. However, the mechanism underlying these differences remains unclear.

**Figure 1:**
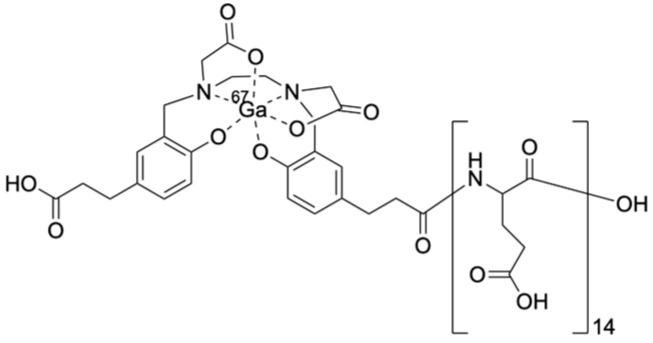
A chemical structure of [^67^Ga]Ga-HBED-CC-(Glu)_14_. [^67^Ga]Ga-HBED-CC-(l-Glu)_14_, [^67^Ga]Ga-HBED-CC-(d-Glu)_14_, [^67^Ga]Ga-HBED-CC-(dl-Glu)_14_, and [^67^Ga]Ga-HBED-CC-(d-Glu-l-Glu)_7_ are stereoisomers of [^67^Ga]Ga-HBED-CC-(Glu)_14_.

In this study, to elucidate the effect of stereoisomers of Glu in radiogallium complex-conjugated (Glu)_n_ on their accumulation in the kidney and to find a strategy to reduce the renal accumulation in some peptide-based radiolabeled compounds, the biodistribution of [^67^Ga]Ga-HBED-CC-(l-Glu)_14_, [^67^Ga]Ga-HBED-CC-(d-Glu)_14_, [^67^Ga]Ga-HBED-CC-(dl-Glu)_14_ (which is composed of racemic Glu), and [^67^Ga]Ga-HBED-CC-(d-Glu-l-Glu)_7_ (which is composed of repeated d-Glu and l-Glu) was compared. The structure of them is shown in Figure 1. Although we were interested in ^68^Ga (t_1/2_ = 68 min) for PET imaging, ^67^Ga (t_1/2_ = 3.3 days) was used in this essential study as an alternative radionuclide because of its long half-life.

## 2. Results

### 2.1. Synthesis of [^67^Ga]Ga-HBED-CC-(d-Glu)_14_, [^67^Ga]Ga-HBED-CC-(dl-Glu)_14_, and [^67^Ga]Ga-HBED-CC-(d-Glu-l-Glu)_7_

All [^67^Ga]Ga-HBED-CC-conjugated Glu peptides were synthesized with radiochemical yields greater than 95%; thus, they were used for further experiments without purification as having a radiochemical purity > 95%. Their radiochromatograms are shown in Figure S1.

### 2.2. Hydroxyapatite-Binding Assay

**Figure 2:**
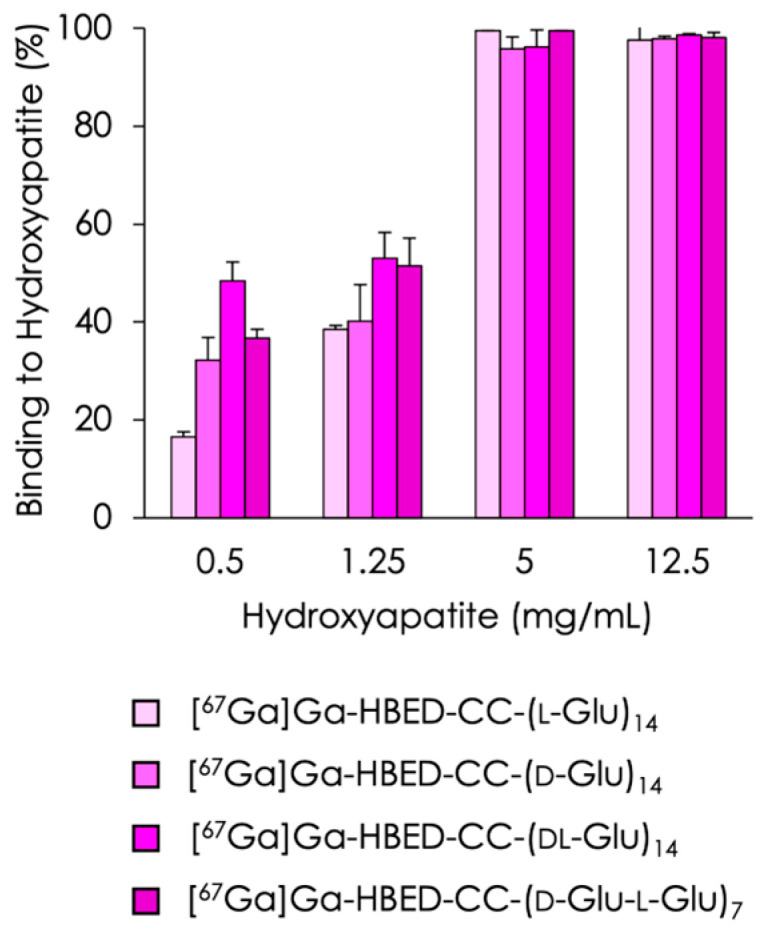
Binding ratios of [^67^Ga]Ga-HBED-CC-(l-Glu)_14_, [^67^Ga]Ga-HBED-CC-(d-Glu)_14_, [^67^Ga]Ga-HBED-CC-(dl-Glu)_14_, and [^67^Ga]Ga-HBED-CC-(d-Glu-l-Glu)_7_ to hydroxyapatite beads. Binding of each [^67^Ga]Ga-HBED-CC-(Glu)_14_ to hydroxyapatite beads increased with the amount of hydroxyapatite. Data are expressed as the mean ± SD for four samples. Data of [^67^Ga]Ga-HBED-CC-(l-Glu)_14_ from reference [22].

To determine the affinity of the compounds for hydroxyapatite, the hydroxyapatite-binding assay was performed. The hydroxyapatite-binding rates of [^67^Ga]Ga-HBED-CC-(l-Glu)_14_, [^67^Ga]Ga-HBED-CC-(d-Glu)_14_, [^67^Ga]Ga-HBED-CC-(dl-Glu)_14_, and [^67^Ga]Ga-HBED-CC-(d-Glu-l-Glu)_7_ are illustrated in Figure 2. The binding profiles of each tracer were similar.

### 2.3. Biodistribution Experiments

**Figure 3:**
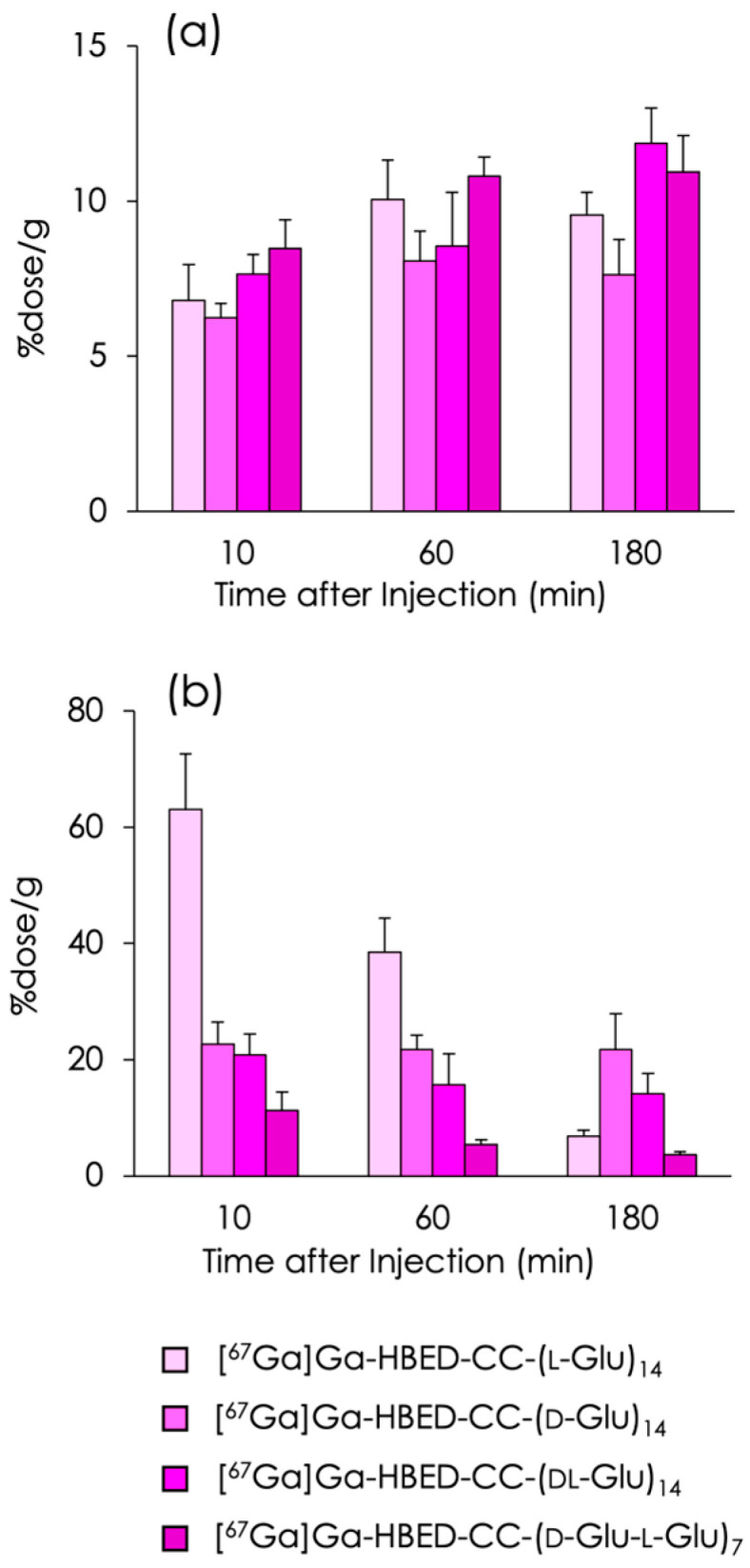
Radioactivity in (**a**) bone and (**b**) kidney after intravenous injection of [^67^Ga]Ga-HBED-CC-(l-Glu)_14_, [^67^Ga]Ga-HBED-CC-(d-Glu)_14_, [^67^Ga]Ga-HBED-CC-(dl-Glu)_14_, and [^67^Ga]Ga-HBED-CC-(d-Glu-l-Glu)_7_ in normal mice. Each value represents the mean (SD) for four animals. Data of [^67^Ga]Ga-HBED-CC-(l-Glu)_14_ from reference [22].

The accumulations of [^67^Ga]Ga-HBED-CC-(l-Glu)_14_, [^67^Ga]Ga-HBED-CC-(d-Glu)_14_, [^67^Ga]Ga-HBED-CC-(dl-Glu)_14_, and [^67^Ga]Ga-HBED-CC-(d-Glu-l-Glu)_7_ in the bones and kidneys of normal mice are reported in Figure 3, and detailed data pertaining to their biodistribution are displayed in Tables S1–S3 and a previous paper [22]. All ^67^Ga-labeled peptides accumulated and were retained in the bone at high levels, and their accumulation rates were similar (Figure 3a). In turn, the renal accumulation of the tracers was largely dependent on the optical isomer of the constituent amino acid, Glu (Figure 3b). In all [^67^Ga]Ga-HBED-CC-(Glu)_14_ compounds, radioactivity in all tissues except the bones and kidneys was low (Tables S1–S3).

## 3. Discussion

Generally, peptides composed of d-type amino acids are more stable in vivo than peptides composed of l-type amino acids because peptidases recognize and metabolize the latter [26]. Therefore, studies have often been performed to improve the stability of peptides by replacing l-amino acids with d-amino acids [27]. In our previous study, repeated d-Asp, i.e., (d-Asp)_n_, was initially synthesized in an attempt to achieve a higher bone accumulation compared to (l-Asp)_n_ as bone-targeting carriers. However, the accumulation of [^67^Ga]Ga-DOTA-(d-Asp)_n_ and [^67^Ga]Ga-DOTA-(l-Asp)_n_ in bone was hardly different because the two types of tracers showed extremely rapid clearance from the blood [21]. Similarly, in the present study, [^67^Ga]Ga-HBED-CC-(l-Glu)_14_, [^67^Ga]Ga-HBED-CC-(d-Glu)_14_, [^67^Ga]Ga-HBED-CC-(dl-Glu)_14_, and [^67^Ga]Ga-HBED-CC-(d-Glu-l-Glu)_7_ exhibited a similar bone accumulation (Figure 3) caused by rapid clearance and a similar affinity for hydroxyapatite as expected.

Several studies have investigated the differences in the renal accumulation of peptides composed of l-amino acids and d-amino acids. One study reported that the antimicrobial peptide composed of d-amino acid named danalexin resulted in prolonged retention in the rat kidney compared with the counterpart peptide, ranalexin, which is composed of l-amino acid [28]. That article indicates that the observed prolonged retention is attributable to the increased stability of the peptide and its increased resistance to peptidolysis. In turn, the initial renal uptake of d-amino acid peptides compared with l-amino acid peptides tends to be lower. This difference is primarily caused by the altered stereochemistry of d-amino acids, which affects their recognition and transport by renal uptake mechanisms, such as specific transporters and receptors [29]. In this study, [^67^Ga]Ga-HBED-CC-(l-Glu)_14_ exhibited a very high initial uptake into the kidney, with the accumulation gradually decreasing thereafter in a time-dependent manner. In contrast, [^67^Ga]Ga-HBED-CC-(d-Glu)_14_ showed a moderate uptake in the kidney and the accumulation was retained. These results are consistent with the above-mentioned descriptions provided in previous articles. 

[^67^Ga]Ga-HBED-CC-(d-Glu-l-Glu)_7_ was synthesized as a peptide in which the d-Glu and l-Glu were alternately repeated. [^67^Ga]Ga-HBED-CC-(d-Glu-l-Glu)_7_ exhibited the lowest radioactivity in the kidney among all [^67^Ga]Ga-HBED-CC-(Glu)_14_ compounds, although the explanation for this observation remains unknown. It is known that poly-l-glutamic acid could be α-helical or have random coil peptide conformation depending on conditions [30]. The presence of d-type amino acids induces conformational preferences not followed by peptides consisting of naturally abundant l-type amino acids [31]. Therefore, we assume that repeating the d-Glu and l-Glu may have a very different conformation from oligo-l-glutamic acid, making it less susceptible to transporter recognition for kidney uptake. This is only speculation; further studies are needed to elucidate the mechanism. 

Because [^67^Ga]Ga-HBED-CC-(d-Glu-l-Glu)_7_ showed an equivalent bone accumulation compared with the remaining [^67^Ga]Ga-HBED-CC conjugated (Glu)_14_ compounds, [^67^Ga]Ga-HBED-CC-(d-Glu-l-Glu)_7_ possessed the most ideal pharmacokinetics as a bone-seeking radiopharmaceutical. Although the compounds assessed in this study targeted hydroxyapatite in the bone and, therefore, no difference in target directivity should exist between d-Glu and l-Glu, the d- and l-peptides may not be easily interchangeable when targeting receptors and enzymes, etc. This is the reason for the limited number of targets that can be used. If target directivity is maintained, repeated d- and l-peptides may be a useful method for reducing the renal accumulation of peptides. However, the reduction of renal uptake using repeated d- and l-peptides has only been confirmed for Glu-peptides in this study. Therefore, future studies are needed to determine whether this strategy can be applied to other peptides.

A typical method of reducing the renal accumulation of radiopharmaceuticals includes coinfusion of lysine and arginine [32]. This method is used in clinical [^177^Lu]Lu-DOTATATE for radiation protection of kidneys [33]. Administration of the mixture of lysine and arginine is a relatively safe and effective method. However, the long time required to administer the mixture of lysine and arginine and its high dosage volume are problems. Another method to reduce renal radioactivity is introducing a cleavable linkage by enzymes on the renal brush border membrane [34,35]. This method is an excellent scientific approach; however, the drug design is complex. The method suggested in this study may overcome these problems. However, as mentioned, it is expected to be used in limited cases. It may be an option for reducing renal accumulation of radiopharmaceuticals.

A [^67^Ga]Ga-HBED-CC-(dl-Glu)_14_ peptide composed of racemic glutamic acid was also synthesized and evaluated. [^67^Ga]Ga-HBED-CC-(dl-Glu)_14_ exhibited a similar kidney uptake to [^67^Ga]Ga-HBED-CC-(d-Glu)_14_, and its radioactivity decreased gradually. As [^67^Ga]Ga-HBED-CC-(dl-Glu)_14_ is a mixture of various isomers, it was difficult to assess the relationship between its structure and renal radioactivity. It seems to be reasonable to assume that the radioactivity of [^67^Ga]Ga-HBED-CC-(dl-Glu)_14_ in the kidney was intermediate compared with the remaining [^67^Ga]Ga-HBED-CC-(Glu)_14_ compounds.

[^67^Ga]Ga-DOTA-(l-Glu)_14_ also showed a very high initial uptake in the kidney, and its accumulation was retained in a previous report [20]. The difference in retention observed between [^67^Ga]Ga-HBED-CC-(l-Glu)_14_ and [^67^Ga]Ga-DOTA-(l-Glu)_14_ may have been depended on the difference in the lipophilicity of their complexes. In turn, the renal accumulation and retention of [^67^Ga]Ga-DOTA-(l-Asp)_14_ and [^67^Ga]Ga-DOTA-(d-Asp)_14_ were not high and were similar. The mechanisms of uptake of oligo-aspartic acid and oligo-glutamic acid may be different.

## 4. Materials and Methods

### 4.1. Materials

Electrospray ionization mass spectra (ESI-MS) were obtained with a JEOL JMS-T100TD (JEOL Ltd., Tokyo, Japan). Purification of peptides was performed using an HPLC system (LC-20AD pump, SPD-20A UV detector at a wavelength of 220 nm, and CTO-20A column oven maintained at 40 °C; Shimadzu, Kyoto, Japan). Thin layer chromatography (TLC) analyses were performed with silica plates (Art 5553, Merck, Darmstadt, Germany). Fmoc-l-Glu(O*t*Bu)-OH and Fmoc-d-Glu(O*t*Bu)-OH were purchased from AmBeed (Arlington Heights, IL, USA). 2-Chlorotrityl chloride resin was purchased from Watanabe Chemical Industries, Ltd. (Hiroshima, Japan). *N*,*N*-Diisopropylethylamine (DIEA) was purchased from Nacalai Tesque (Kyoto, Japan). 1,3-Diisopropylcarbodiimide (DIPCDI) and 1-hydroxybenzotriazole hydrate (HOBt) were purchased from Kokusan Chemical Co., Ltd. (Tokyo, Japan). Other reagents were of reagent grade and used as received.

### 4.2. Synthesis of HBED-CC-(d-Glu)_14_, HBED-CC-(dl-Glu)_14_, and HBED-CC-(d-Glu-l-Glu)_7_

HBED-CC-(d-Glu)_14_, HBED-CC-(dl-Glu)_14_, and HBED-CC-(d-Glu-l-Glu)_7_ were synthesized according to a previous report [22]. Namely, the protected peptidyl resin was manually constructed using an Fmoc-based solid-phase methodology based on a previously described method [25]. After the construction of the peptide chain on the resin, the Fmoc protecting group was removed using 20% piperidine in DMF. Subsequently, a mixture containing 2.5 equivalents of HBED-CC-tris(tert-butyl) ester (which was synthesized according to the previous report [36]), DIPCDI, and HOBt in DMF was added and allowed to react at room temperature for 1.5 h. After the cleavage of the peptides from the resin and the deprotection using triisopropylsilane and trifluoroacetic acid (TFA) (5:95), the crude products were purified using reversed-phase (RP) HPLC on a Cosmosil 5C_18_-AR-II column (10 × 150 mm; Nacalai Tesque) at a flow rate of 4 mL/min with a gradient mobile phase of 20–60% methanol in water containing 0.1% TFA over 20 min, respectively. Chromatograms were obtained by monitoring the UV absorption at a wavelength of 220 nm. The fractions containing HBED-CC-(d-Glu)_14_, HBED-CC-(dl-Glu)_14_, and HBED-CC-(d-Glu-l-Glu)_7_ were determined by mass spectrometry and collected. The solvent was removed by lyophilization to provide HBED-CC conjugated Glu peptides as white powders.

MS (ESI+) analysis of HBED-CC-(d-Glu)_14_ calcd for C_96_H_132_N_16_O_52_ [M + 2H]^2+^: *m*/*z* = 1170.9 found 1170.8, yield: 3%.
MS (ESI+) analysis of HBED-CC-(dl-Glu)_14_ calcd for C_96_H_132_N_16_O_52_ [M + 2H]^2+^: *m*/*z* = 1170.9 found 1170.9, yield: 3%.
MS (ESI+) analysis of HBED-CC-(d-Glu-l-Glu)_7_ calcd for C_96_H_132_N_16_O_52_ [M + 2H]^2+^: *m*/*z* = 1170.9 found 1170.7, yield: 1%.

### 4.3. Preparation of [^67^Ga]Ga-HBED-CC-(d-Glu)_14_, [^67^Ga]Ga-HBED-CC-(dl-Glu)_14_, and [^67^Ga]Ga-HBED-CC-(d-Glu-l-Glu)_7_

[^67^Ga]Ga-citrate was purchased from Nihon Medi-Physics Co., Ltd. (Tokyo, Japan), and converted [^67^Ga]GaCl_3_ by using Sep-Pak^®^ Silica Plus Light Cartridge (Waters Co., Ltd., Milford, MA, USA) according to a previous report [37,38]. Approximately 50 μg of HBED-CC-(d-Glu)_14_, HBED-CC-(dl-Glu)_14_, or HBED-CC-(d-Glu-l-Glu)_7_ was dissolved in 80 μL of 1 M *N*-2-hydroxyethylpiperazine-*N*-2-ethanesulfonic acid (HEPES) solution (pH 5.0), and 20 μL of [^67^Ga]GaCl_3_ solution in 0.1 M HCl was added and allowed to react at 80 °C for 10 min. The radiochemical purities of ^67^Ga-HBED-CC conjugated Glu peptides were determined via TLC analyses using silica plates with acetonitrile and H_2_O (1:1) as a developing solvent. RP-HPLC was performed with a Cosmosil 5C_18_-II column (4.6 × 150 mm) at a flow rate of 1 mL/min with a gradient mobile phase of 20% ethanol in water containing 10 mM tetrabutylammoniumhydroxide (TBAH) to 55% ethanol in water containing 10 mM TBAH for 20 min. 

### 4.4. Hydroxyapatite-Binding Assays

Hydroxyapatite-binding assays were performed according to procedures described previously [7]. Briefly, hydroxyapatite beads (Bio-Gel; Bio-Rad, Hercules, CA, USA) were suspended in Tris/HCl-buffered saline (50 mM, pH 7.4) at 1 mg/mL, 2.5 mg/mL, 10 mg/mL, and 25 mg/mL. For the solutions of [^67^Ga]Ga-HBED-CC-(d-Glu)_14_, [^67^Ga]Ga-HBED-CC-(dl-Glu)_14_, and [^67^Ga]Ga-HBED-CC-(d-Glu-l-Glu)_7_, the ligand concentrations were adjusted to 19.5 µM by adding the corresponding HBED-CC conjugated Glu peptides. A solution of each [^67^Ga]Ga-HBED-CC-(d-Glu)_14_, [^67^Ga]Ga-HBED-CC-(dl-Glu)_14_, and [^67^Ga]Ga-HBED-CC-(d-Glu-l-Glu)_7_ (200 µL each) was added to 200 μL of the hydroxyapatite suspension, and the samples were gently shaken for 1 h at room temperature. After centrifugation at 10,000× *g* for 5 min, the radioactivity of the supernatants was measured using an auto well gamma counter (ARC-7010B, ALOKA Co., Ltd., Tokyo, Japan). Control experiments were performed using the same procedure without hydroxyapatite beads, which resulted in an adsorption of radioactivity to vials of less than 0.1%. The ratios of binding were determined as follows:

Hydroxyapatite binding (%) = (1 − [sample supernatant radioactivity]/[control supernatant radioactivity]) × 100

### 4.5. Animals

Experiments with animals were conducted in strict accordance with the Guidelines for the Care and Use of Laboratory Animals of Kanazawa University. The animal experimental protocols used were approved by the Committee on Animal Experimentation of Kanazawa University (AP-204165, 5 April 2023). The animals were housed with free access to food and water at 23 °C with a 12 h alternating light/dark schedule.

### 4.6. Biodistribution Experiments

Biodistribution experiments were performed after an intravenous administration of each tracer solution diluted in saline (37 kBq/100 μL) to 6-week-old male ddY mice (weight, 27–32 g, Japan SLC, Inc., Hamamatsu, Japan). Four mice were sacrificed at each time point for each compound at 10, 60, and 180 min post-injection. The tissues of interest were dissected and weighed. Complete left femurs were isolated as representative bone samples, radioactivity counts were determined using an auto well gamma counter (ARC-7010, ALOKA Co., Ltd.), and counts were corrected for the background radiation and physical decay that occurred during counting.

## 5. Conclusions

In this study, we compared [^67^Ga]Ga-HBED-CC-(l-Glu)_14_, [^67^Ga]Ga-HBED-CC-(d-Glu)_14_, [^67^Ga]Ga-HBED-CC-(dl-Glu)_14_, and [^67^Ga]Ga-HBED-CC-(d-Glu-l-Glu)_7_. In the case of radiopharmaceuticals that use (Glu)_14_ as a bone-directing peptide, the optical isomerism of the constituent amino acids resulted in comparable bone affinity, whereas significant differences in the kidney uptake and retention were observed. Among the [^67^Ga]Ga-HBED-CC complex-conjugated–(Glu)_14_ compounds, [^67^Ga]Ga-HBED-CC-(d-Glu-l-Glu)_7_ exhibited the lowest kidney accumulation. The conformation of repeating d-Glu and l-Glu may differ significantly from that of oligo-l-glutamic acid, potentially reducing its recognition by transporters for kidney uptake. Although further studies are needed to elucidate the mechanism, in some cases, the strategy of using repeated d- and l-peptides may be a useful method for reducing their renal accumulation.

## Data Availability

The original contributions presented in the study are included in the article/Supplementary Material, further inquiries can be directed to the corresponding author.

## Supplementary Materials

The following supporting information can be downloaded at: https://www.mdpi.com/article/10.3390/molecules29173993/s1, Figure S1: HPLC radiochromatograms of ^67^Ga-labeled compounds; Table S1: Biodistribution of [^67^Ga]Ga-HBED-CC-(d-Glu)_14_; Table S2: Biodistribution of [^67^Ga]Ga-HBED-CC-(dl-Glu)_14_; Table S3: Biodistribution of [^67^Ga]Ga-HBED-CC-(d-Glu-l-Glu)_7_.
