# Supplementary material for: Differences in the Renal Accumulation of Radiogallium-Labeled (Glu)14 Peptides Containing Different Optical Isomers of Glutamic Acid
Source: Molecules. 2024 Aug 23;29(17):3993. doi: 10.3390/molecules29173993 (PMC11396517; doi:10.3390/molecules29173993)
Supplement: Supplementary file 1 [file molecules-29-03993-s001.zip › molecules-3149960-SI.pdf]

Supporting Information

Differences in the renal accumulation of radiogallium-labeled $(Glu)_{14}$ peptides containing different optical isomers of glutamic acid

Kazuma Ogawa[1,2*], Kota Nishizawa[2], Kenji Mishiro[1], Masayuki Munekane[2], Takeshi Fuchigami[2], Hiroaki Echigo[2], Hiroshi Wakabayashi[3], Seigo Kinuya[3]

[1]Kanazawa University, Institute for Frontier Science Initiative, Kakuma-machi, Kanazawa, Ishikawa 920-1192, Japan;

[2]Graduate School of Medical Sciences, Kanazawa University, Kakuma-machi, Kanazawa, Ishikawa 920-1192, Japan;

[3]Department of Nuclear Medicine, Institute of Medical, Pharmaceutical and Health Sciences, Kanazawa University, Takara-machi 13-1, Kanazawa, Ishikawa 920-8641, Japan

***Corresponding Author**

Institute for Frontier Science Initiative; Kanazawa University; Kakuma-machi, Kanazawa 920-1192; Japan.

Telephone: 81-76-234-4460; Fax: 81-76-234-4460

Table of contents

Figure S1 HPLC radiochromatograms of ^{67}Ga-labeled compounds S3

Table S1 Biodistribution of [^{67}Ga]Ga-HBED-CC-(D-Glu)$_{14}$ S4

Table S2 Biodistribution of [^{67}Ga]Ga-HBED-CC-(DL-Glu)$_{14}$ S5

Table S3 Biodistribution of [^{67}Ga]Ga-HBED-CC-(D-Glu-L-Glu)$_7$ S6

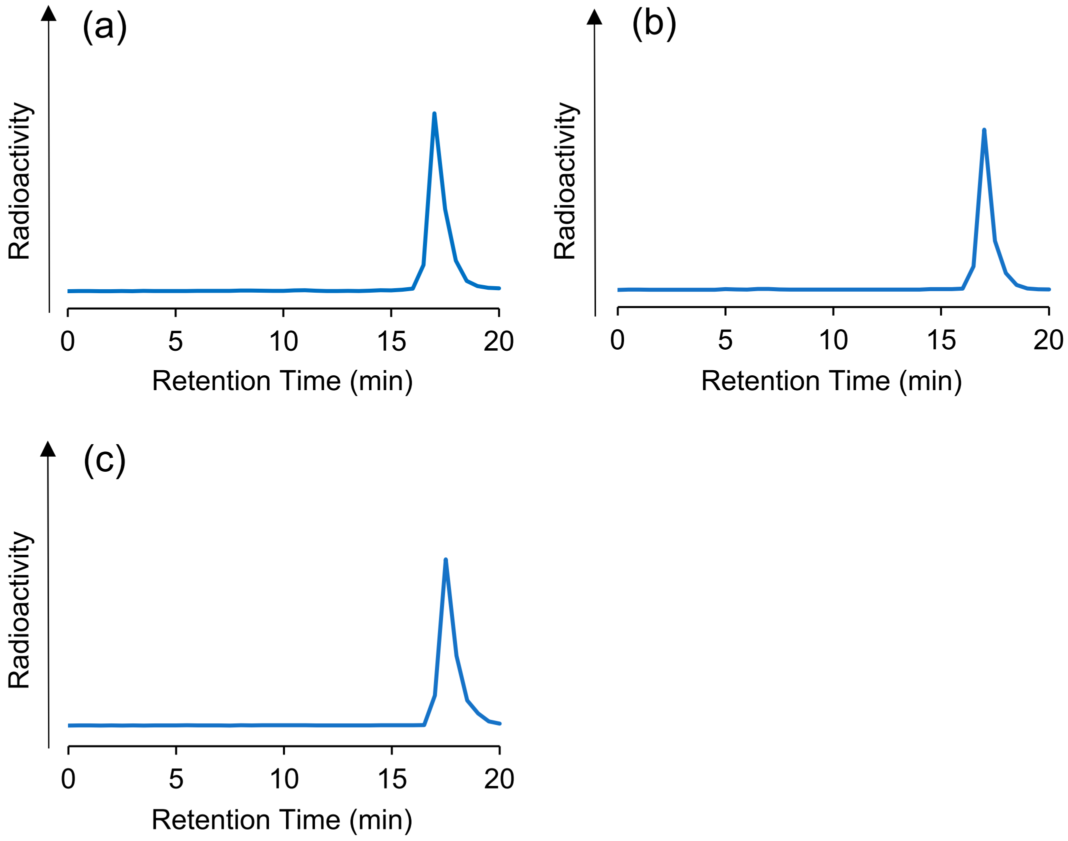

Figure S1. HPLC radiochromatograms of (a) [^{67}Ga]Ga-HBED-CC-(D-Glu)$_{14}$, (b) [^{67}Ga]Ga-HBED-CC-(DL-Glu)$_{14}$, and (c) [^{67}Ga]Ga-HBED-CC-(D-Glu-L-Glu)$_{7}$

Table S1. Biodistribution of radioactivity after intravenous administration of [^{67}Ga]Ga-HBED-CC-(D-Glu)$_{14}$ in normal mice[a]

Tissue	Time after administration		
	10 min	60 min	180 min
Blood	1.80 (0.22)	0.12 (0.01)	0.01 (0.00)
Liver	0.47 (0.03)	0.41 (0.14)	0.20 (0.05)
Kidney	22.68 (3.74)	21.70 (2.52)	21.77 (6.17)
Small-intestine	0.53 (0.12)	0.26 (0.05)	0.57 (0.25)
Large-intestine	0.60 (0.39)	0.10 (0.01)	0.81 (0.40)
Spleen	0.31 (0.02)	0.11 (0.05)	0.03 (0.01)
Pancreas	0.54 (0.10)	0.14 (0.04)	0.09 (0.01)
Lung	1.37 (0.24)	0.20 (0.04)	0.07 (0.03)
Heart	0.69 (0.13)	0.14 (0.02)	0.09 (0.02)
Stomach[b]	0.57 (0.38)	0.51 (0.42)	0.76 (0.33)
Bone (Femur)	6.23 (0.48)	8.07 (0.95)	7.62 (1.14)
Muscle	0.64 (0.05)	0.09 (0.05)	0.06 (0.03)
Brain	0.04 (0.01)	0.01 (0.00)	0.00 (0.00)
F/B ratio[c]	3.49 (0.35)	69.98 (6.63)	1447.63 (652.91)

[a] Expressed as % injected dose. Each value represents the mean (SD) for four animals.

[b] Expressed as % injected dose

[c] Femur:blood ratio

Table S2. Biodistribution of radioactivity after intravenous administration of [^{67}Ga]Ga-HBED-CC-(DL-Glu)$_{14}$ in normal mice[a]

Tissue	Time after administration		
	10 min	60 min	180 min
Blood	1.81 (0.18)	0.13 (0.04)	0.09 (0.02)
Liver	0.76 (0.15)	0.32 (0.07)	0.40 (0.06)
Kidney	20.76 (3.66)	15.70 (5.32)	14.15 (3.48)
Small-intestine	0.49 (0.02)	0.51 (0.33)	0.24 (0.04)
Large-intestine	0.46 (0.06)	0.14 (0.08)	0.50 (0.15)
Spleen	0.44 (0.03)	0.12 (0.02)	0.10 (0.02)
Pancreas	0.81 (0.55)	0.18 (0.06)	0.14 (0.03)
Lung	1.57 (0.16)	0.20 (0.03)	0.17 (0.04)
Heart	0.68 (0.06)	0.13 (0.05)	0.11 (0.04)
Stomach[b]	0.58 (0.38)	1.34 (1.97)	0.19 (0.16)
Bone (Femur)	7.64 (0.64)	8.55 (1.73)	11.86 (1.15)
Muscle	0.63 (0.17)	0.11 (0.04)	0.12 (0.05)
Brain	0.06 (0.03)	0.02 (0.02)	0.04 (0.06)
F/B ratio[c]	4.26 (0.54)	68.87 (10.55)	136.37 (17.12)

[a] Expressed as % injected dose. Each value represents the mean (SD) for four animals.

[b] Expressed as % injected dose

[c] Femur:blood ratio

Table S3. Biodistribution of radioactivity after intravenous administration of [^{67}Ga]Ga-HBED-CC-(D-Glu-L-Glu)$_7$ in normal mice[a]

Tissue	Time after administration		
	10 min	60 min	180 min
Blood	1.83 (0.37)	0.09 (0.04)	0.01 (0.01)
Liver	0.46 (0.03)	0.19 (0.02)	0.19 (0.03)
Kidney	11.20 (3.26)	5.36 (0.89)	3.63 (0.48)
Small-intestine	0.45 (0.08)	1.02 (0.65)	1.02 (0.46)
Large-intestine	0.36 (0.07)	0.09 (0.01)	0.92 (0.51)
Spleen	0.42 (0.06)	0.04 (0.01)	0.02 (0.02)
Pancreas	0.62 (0.04)	0.13 (0.05)	0.09 (0.02)
Lung	1.51 (0.38)	0.13 (0.02)	0.06 (0.02)
Heart	0.80 (0.05)	0.16 (0.05)	0.11 (0.01)
Stomach[b]	0.32 (0.03)	0.84 (0.69)	0.48 (0.19)
Bone (Femur)	8.48 (0.91)	10.80 (0.61)	10.94 (1.18)
Muscle	0.62 (0.20)	0.09 (0.02)	0.10 (0.04)
Brain	0.07 (0.02)	0.01 (0.01)	0.01 (0.00)
F/B ratio[c]	4.71 (0.56)	141.41 (80.75)	1491.58 (841.65)

[a] Expressed as % injected dose. Each value represents the mean (SD) for four animals.

[b] Expressed as % injected dose

[c] Femur:blood ratio
